# A quasi‐experimental study on the effect of health and food safety training intervention on restaurant food handlers during the COVID‐19 pandemic

**DOI:** 10.1002/fsn3.2326

**Published:** 2021-05-20

**Authors:** Fatemeh Mohammadi‐Nasrabadi, Yeganeh Salmani, Fatemeh Esfarjani

**Affiliations:** ^1^ Research Department of Food and Nutrition Policy and Planning Faculty of Nutrition Sciences and Food Technology, National Nutrition & Food Technology Research Institute (NNFTRI), Shahid Beheshti University of Medical Sciences Tehran Iran

**Keywords:** COVID‐19 prevention, food handlers, public health, training intervention

## Abstract

The restaurant business has turned into a dynamic and ever‐growing industry. So, food safety must be a priority for these establishments, especially during the COVID‐19 pandemic. The aim of this study was to determine the effect of training intervention on the health and food safety knowledge, attitude, and self‐reported practice (KAP) of restaurant food handlers during the COVID‐19 pandemic. This quasi‐experimental study was conducted on 159 restaurant food handlers in Tehran, Iran. The training intervention was developed based on the latest global guidelines. The KAP of the subjects was measured before and after the training. Fisher's exact test, paired *t* test, and repeated measures ANOVA were used for statistical analysis. Data analysis was done using the IBM_SPSS software. The total knowledge scores of participants were low (17.6%), moderate (35.2%), and good (47.2%) before training, which were changed to 5% (low), 23.9% (moderate), and 71.1% (good) after training. The total pretraining attitude scores were 0.6, 77.4, 18.2, and 3.8% that were changed to 0% (strongly negative), 49.1% (negative), 33.3% (positive), and 17.6% (strongly positive), respectively. Also, the self‐reported practice scores of the participants before training were 1.3, 56, and 42.7 that were changed to 0% (weak), 26.4% (acceptable), and 73.6% (desirable) after the intervention, respectively. Paired *t* test results showed a statistically significant increase in all scores. The interaction of training with age and education was statistically significant in increasing the knowledge and attitude scores of the participants by the repeated measures ANOVA. Improving the KAP of food handlers by health and food safety training can improve the status of restaurants and minimize the outbreak of pandemic diseases, including COVID‐19, which is an effective step in community health. Thus, it is an urgent need for policymakers to design an online system of continuous food safety training for food handlers.

## Graphical abstract

This study aimed to determine the efficacy of health and food safety training in food services during the COVID‐19 pandemic.The present study revealed a significant improvement in the knowledge, attitude, and practice of food‐handlers after the health and food safety training that can minimize the COVID‐19 outbreaks. Accordingly, public health authorities should prioritize food‐handlers in the restaurants for training courses.

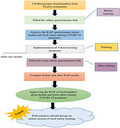

## 1 INTRODUCTION

Over the past two decades, the changing lifestyle of people all over the world has reduced the consumption of home foods, and food consumption outside has grown significantly (Bozoglu et al., 2013). Therefore, the restaurant business has turned into a very dynamic and ever‐growing industry, and so, food safety must be a top priority for these establishments, especially during the COVID‐19 pandemic (Angulo & Jones, 2006).

At the time being, with the emergence of the COVID‐19 pandemic, the restaurant industry is struggling to organize itself and strives to protect the health of both food producers and food consumers, because it is not possible in the this industry to work from long distances, and the staffs are forced to continue working in the former work environment ( World Health Organization, 2020).

COVID‐19 is not a foodborne virus and cannot survive or thrive in food, but one can be infected when handling food without proper hygiene and precautions, just as when handling any other item that has come into contact with a person infected by coronavirus. Also, the heating process for at least 30 min at 60°C is effective in killing the viruses like SARS ( Bogoch et al., 2020; Duda‐Chodak et al., 2020; Feng, 2020; Jain, 2020).

Coronavirus was detected in saliva (Baier et al., (2020)), so food handlers can spread the virus by coughing, speaking, breathing, sneezing, or singing. All these activities can create an infectious aerosol when it contains pathogens, which are projected into the surrounding air. It is suspected that exhaled air from both the nose and mouth is able to mix with air in the breathing zone of other persons standing nearby, including customers and staff in the restaurants (Tang et al., 2006).

**FIGURE 1:**
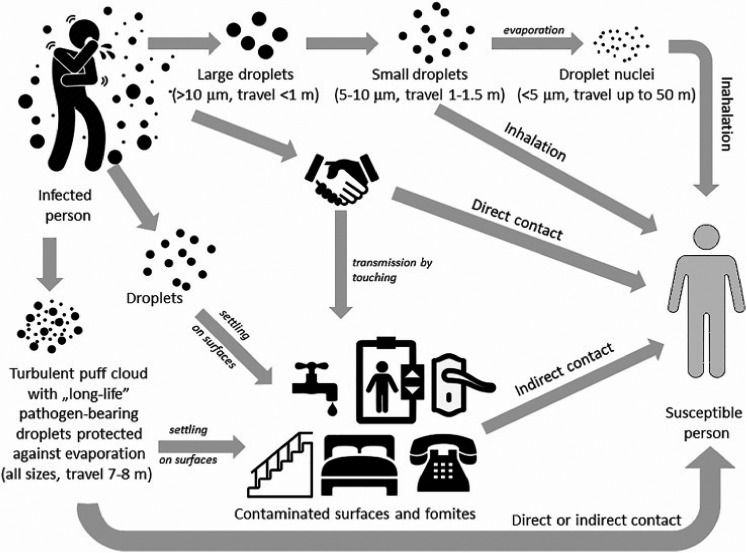
Transmission routes involving direct and, indirect contact by contaminated surfaces and fomites, as well as by droplets (short range) and droplet nuclei (long range) spreading (10)

The virus is transmitted directly through contact with an infected person's body fluids or indirectly through contact with surfaces contaminated with coughing or sneezing particles. Therefore, food handlers must carefully observe hygiene items, especially hand hygiene and wearing mask (Kingdom GotU, 2020) (Figure 1). It is also important to closely monitor the health of food handling staff and identify all infected people, especially asymptomatic carriers of the virus, thereby preventing its spread (Feng, 2020).

Any level of mishandling can contribute to the occurrence or spread of COVID‐19 outbreaks. It is critical that food handlers be trained properly to increase food safety and share the long‐term benefits within the food industry (Egan et al., 2007). With continuous practical training, the prevalence of risks from eating contaminated foods can be minimized (Chapman et al., 2010; Naing et al., 2007; Soon et al., 2012). Proper health and food safety training is essential to decrease the incidence and overall rate of COVID‐19 outbreak (Olaimat et al., 2020).

Due to the fact that no research has so far been done in this field in Iran, and there are limited monitoring programs on how to provide health and food safety in restaurants (Ali, 2015), in the midst of the global COVID‐19 pandemic, with the restaurant industry disproportionately negatively affected, this study examines Iranian restaurant food handlers’ pre‐and post‐training in order to provide a few guidelines as conditions evolve. To our knowledge, this is the first study regarding the effect of health and food safety training intervention on restaurant food handlers during the COVID‐19 pandemic.

## 2 MATERIALS AND METHODS

### 2.1 Study design

A quasi‐experimental study was designed to assess the impact of a training intervention on the health and food safety knowledge, attitude, and self‐reported practice of restaurant food handlers during the COVID‐19 pandemic.

### 2.2 Questionnaire design

The questionnaire of training intervention was developed based on the latest global guidelines, the instructions set by Iran's Ministry of Health and Education (MOH), and literature reviews, as well as the WHO, CDC, and other guidelines in the field of health and food safety during the COVID‐19 pandemic in restaurants and food services (Bogoch et al., 2020; Center of disease control and prevention(CDC), 2020; Duda‐Chodak et al., 2020; Kingdom GotU, 2020; Olaimat et al., 2020; Ministry of Health & Medical Education, 2020; Europian commissin directorate‐general for health & food safety, 2020; Mohammadi‐Nasrabadi et al., 2020; Food Standards Agency, 2020; Esfarjani & Salmani, 2020).

All items with a Cronbach's α of >0.7 were included in the instrument (Gliem & Gliem, 2013). Test–retest reliability was also assessed to check the stability of the questionnaire. Restaurant owners and food handlers were informed about the study goal and agreed to participate in the study (*n* = 10) were recruited to complete the questionnaire at two time points 2 weeks apart with no food safety training intervening. Cranach's alpha was used to test the reliability of the questionnaire along with the test–retest method for testing reliability (0.72) and intraclass correlation coefficient (0.96). Among the corrective changes made to the questionnaire, four items were adapted and four more items were added. Finally, the total of 19 items remained. The final questionnaire was pilot‐tested with three food handlers who did not attend the study, and their comments and feedbacks, regarding the clarity of the questions were sought. No further item was removed from the final version. The questionnaire is available as a supplementary file.

### 2.3 Population

Twenty restaurants were selected randomly from the existing data (*N* = 50) of a previous project (Esfarjani et al., 2019), twelve of them were willing to attend the study. A number of inclusion and exclusion criteria were considered as well. The inclusion criteria comprised of all restaurant food handlers working as chef, kitchen assistant or waiter, aged 20 years or above, having access to smartphone and WhatsApp messenger, and also agreeing to participate actively in the educational program. The exclusion criterion was the volunteer's unwillingness to continue with the research for any reason. During this course, no attendee received any formal training on this subject.

### 2.4 Data collection

The online questionnaire link was sent to the restaurant managers, and they were asked to send the online questionnaire link to their food handlers, who filled the questionnaire either on the spot or later at their own convenience. The research objectives were explained to the participants at the beginning before completion of the survey. One hundred and ninety two participants filled out the form, of which eight were not eligible and 18 of them were unable to attend the intervention course due to personal reasons. Seven participants in five restaurants who missed one or more sessions in the study were excluded as not properly exposed by the intervention or post‐test sessions. Totally, 33 participants were omitted during the pre‐ and post‐training and the remaining 159 subjects continued the study to the end.

An online questionnaire with a total of 83 scores was developed. It was divided into three sections. The first section consisted of four sociodemographic questions, including the participants’ age, gender, education level, and health status.

In the second section, there were 19 multiple‐choice items focused on the knowledge, attitude, and self‐reported practice of the restaurant food handlers about COVID‐19 prevention ( Cunha et al., 2019; Kunadu et al., 2016; Majowicz et al., 2015). The knowledge questions were comprised of four items to be scored as zero score for “false,” and “I don't know” answers and 1 score for “true” answers, which classified the scores as low (<2), moderate ( Angulo & Jones, 2006; World Health Organization, 2020), and good ( Jain, 2020).

The attitude consisted of three items to be rated on five‐point Likert scales: “*completely disagree,*” “*disagree,*” “*probably,*” “*agree,*” and “*strongly agree,*” each weighing 1–5 scores, respectively. The total scores were classified as *strongly negative* (<5), *negative* (5–10),, *positive* (11–12), and *strongly positive* (13–15). Some questions were reversed to diminish the possible bias of giving a single similar response in all the items.

As for self‐reported practice, there were 12 items rated as five‐point Likert‐item questions with the responses of *never*, *rarely*, *sometimes*, *often*, and *always*, each weighing 1–5 scores, respectively. The self‐reported practice scores of them were classified as *weak* (<40), *acceptable* (40–50), and *desirable* (51–60). The observed practices presented a slight correlation with the self‐reported practices in a previous study (Cunha et al., 2019).

The last section included three questions about the participants’ source of information, need for education, and how they can increase their knowledge in this issue. Also, they were asked to comment on (a) *What they most liked about the course*,(b) *How it could be improved,* and (c) *What they will do differently at work as a result of their training*.

### 2.5 Training intervention procedure

Training intervention on health and food safety knowledge, attitude, and practice of food handlers during the COVID‐19 pandemic was offered. It is important to mention that the training was conducted just 14 days after the first sampling.

Educational sessions took place once a week (45–55 min) in their workplace during four subsequent weeks for each restaurant. There were PowerPoint presentation and videos/webinar in the form of lecture, group discussion, and also an educational booklet in this field, which was published by the research team (Esfarjani & Salmani, 2020). The intervention procedure is presented in Table 1.

**TABLE 1:** Training intervention's procedure

Session	Topic	Aim	How	Duration (min)	Lead by^a^
First	Introduction, COVID−19 risk management	To make understand the importance of COVID‐19 prevention	Power point presentation	45	Facilitator
Second	Personal hygiene and social distancing during the COVID−19 pandemic	To make understand the importance of wearing gloves, face mask, and washing hands Keeping customers and staffs safe	Power point presentation and video	50	Coordinator
Third	Food safety and environmental health of restaurant	To make understand the WHO’s five keys to safer food: Keep clean Separate raw and cooked Cook thoroughly Keep food at safe temperatures Use safe water and raw materials Sanitation Delivery system during the COVID‐19 pandemic	Power Point and group discussion	55	Coordinator

Fourth	Conclusion	Provide executive approaches, and give their comments and feedback	Online Webinar	50	Moderator

^a^ The facilitator, coordinator, and moderator were from the research team.

Four weeks after the training intervention and 2 weeks from the last date of training for each restaurant as a washout period, same questionnaire link as a post‐test was sent to participants in order to evaluate the sustainability of education in the training sessions. The participants were given a certificate of attendance on completion of the course.

### 2.6 Statistical analysis

Fully completed questionnaires were extracted from Google Forms and exported to a Microsoft Excel 2016 for cleaning and coding. The relevant information was extracted from the questionnaire, and the cleaned data were exported to the IBM‐SPSS software (IBM Corp. Released 2013. IBM SPSS Statistics for Windows, Version 22.0. Armonk, NY: IBM Corp) and analyzed. Numerical and categorical data were summarized as mean (±*SD*) and frequencies, respectively. Normality of the data was checked by One‐Sample Kolmogorov‐Smirnov's test. Fisher's exact test, paired *t* test, and repeated measures ANOVA. If the data were not normal, their nonparametric equivalents (Wilcoxon and Friedman tests) were applied. Statistical significances were set at *p*‐values <.5.

### 2.7 Ethical approval

This study was approved by the Ethics Committee of National Nutrition & Food Technology Research Institute (NNFTRI), Shahid Beheshti University of Medical Sciences, Tehran, Iran (Grant no. IR.SBMU.RETECH.REC.1399.125, 25013). All respondents provided informed consent and were guaranteed anonymity.

## 3 RESULTS

The characteristics of the participants (shown in Table 2) revealed that most of them were men (73.6%). The age group peaked at 20–30 years, which accounted for 49%. Diploma and high school education were the most educational level (56%) of the participants. None of the subjects had any specific training class in this field during the COVID‐19 pandemic.

**TABLE 2:** Sociodemographic characteristics of the participants by gender (*n* = 159)

Variables	Female *n* (%)	Male *n* (%)	Total *n* (%)	*p*‐value
Age (year)				
20–30	19(24.4)	59(75.6)	78(100)	.500
30–40	15(33.3)	30(66.7)	45(100)	
40–50	7(25.9)	20(74.1)	27 (100)	
50–60	1(11.1)	8(88.9)	9(100)	
Education				
≤Diploma	16(18)	73(82)	89(100)	.001^a^
BSc degree	15(25.5)	44(74.5)	59(100)	
MSc degree	11(100)	0	11(100)	
Source of information on COVID−19				
Ministry of Health and Medical Education	18(27.7)	47(72.3)	65(100)	0.611
Internet and social media	13(33.3)	26(66.7)	39(100)	
Family/Friends	1(33.3)	2(66.7)	3(100)	
TV and Radio	1(12.5)	7(87.5)	8(100)	
Union	9(20.5)	35(79.5)	44(100)	
Total	42(26.4)	117(73.6)	159(100)	

^a^ Significant difference in educational level between males and females by Fisher's exact test at *p* < .001.

**FIGURE 2:**
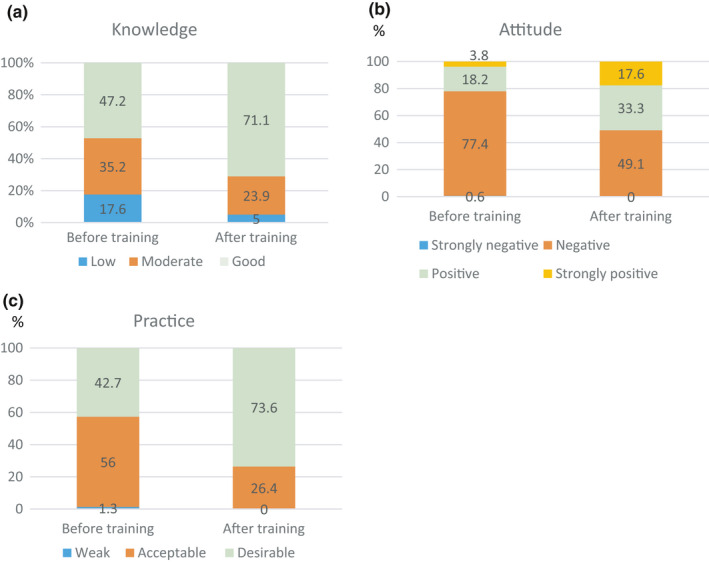
Comparison of the (A) knowledge, (B) attitude, and (C) practice scores’ classification (%) of food handlers before and after training

The results of training intervention on health and food safety during the COVID‐19 pandemic showed that the low, moderate, and good knowledge scores of food handlers before training were 17.6, 35.2, and 47.2%, respectively, that were changed to 5% (low), 23.9% (moderate), and 71.1% (good) after training. The strongly negative, negative, positive, and strongly positive attitude scores of the participants before training were 0.6, 77.4, 18.2, and 3.8%, respectively, which were changed to 0% (strongly negative) 49.1% (negative), 33.3% (positive), and 17.6% (strongly positive) after training. Also, the weak, acceptable, and desirable self‐reported practice scores of the participants before training were 1.3, 56, and 42.7%, respectively, that were changed to 0% (weak), 26.4% (acceptable), and 73.6% (desirable) after training by Fisher's exact test (*p* < .05) (Figure 2).

**FIGURE 3:**
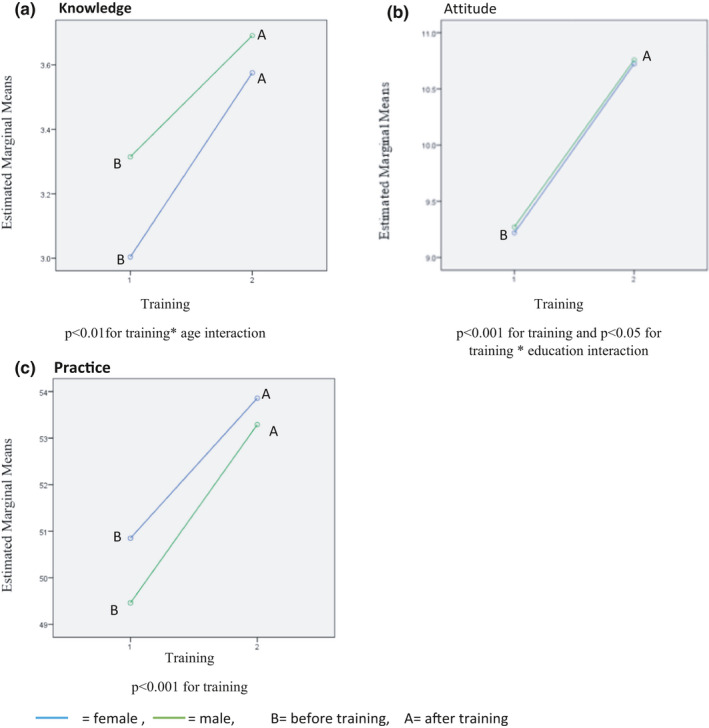
Changes in the (A) knowledge, (B) attitude, and (C) practice scores of food handlers before and after training intervention by gender

The paired *t* test output revealed that the changes in the mean scores of food handlers’ KAP before and after training were statistically significant (*p* < .001). It shows that there is no significant relationship between the knowledge and attitude scores with the gender of food handlers before and after the intervention. The results showed that the self‐reported practice score of females (51.05) before training was significantly higher than that of males (49.39). Also after training, the mean score of females’ self‐reported practice (53.86) was higher than that of males (53.29) but not significant. A significant increase in the attitude and self‐reported practice (but not the knowledge) scores was observed after the training intervention by the repeated measure ANOVA. The interaction of training with age and education was statistically significant in increasing the knowledge and attitude scores of food handlers, respectively (Table 3). It means that age and education can be accounted as two important factors that may affect the training intervention results. In this analysis, time effect, group effect, and interaction between them were assessed; however, because the group effect was not significant, only the other two P‐values were mentioned (Figure 3).

**TABLE 3:** Mean (±*SD*) of knowledge, attitude, and practice scores of food handlers based on their gender before and after the training intervention (*n* = 159)

Variables		Score Mean (±*SD*)		*p*‐value by RMANOVA			
		Before	After	Training	Training*age	Training*education	Training*gender
Knowledge	F^a^	3.07(0.92)	3.57(0.70)	.863	.005	.221	.202
	M^b^	3.29(0.88)	3.69(0.51)				
	Total	3.23(0.89)	3.69(0.57)	<.001^c^			
Attitude	F^a^	9.26(1.68)	10.62(1.62)	<.001	.928	.049	.950
	M^b^	9.26(1.77)	10.79(1.74)				
	Total	9.26(1.74)	10.75(1.70)	<.001^c^			
Practice	F	51.05(4.20)	53.86(4.04)	<.001	.379	.116	.061
	M	49.39(3.88)	53.29(4.01)				
	Total	49.83(4.02)	53.44(4.01)	<.001^c^			

^a^ Female.

^b^ Male.

^c^ Significant difference in total score before and after training by paired‐*t* test (*p* < .001).

## 4 DISCUSSION

The results of the present study showed that there was no gap between knowledge, attitude, and practice. It was seen that the knowledge, attitude, and practice scores increased significantly after training the participants in health and food safety during the COVID‐19 pandemic.

The females’ practice score in health and food safety was higher than in males probably because they are more engaged with food preparation at home, are more multitasking, and pay more attention to COVID‐19. A similar finding was found in a study in Pennsylvania in which the females had higher scores than males. In addition, with increasing age, females tend to do more practice with food handling and food safety issues, and may, therefore, gain higher score than males in studies on adults (Haapala & Probart, 2004).

A brief educational intervention study in Brighton in 2014 on the restaurant food handlers’ knowledge of food allergy showed improvement in their absolute knowledge and practice change (Bailey et al., 2014). Intervention in food safety and hygiene affected the food safety knowledge, behavior, and habits of employees in food and beverage departments of hotels and restaurants in Turkey tourism industry, too (Sanli̇er et al., 2020). Interestingly, a few other studies showed that there may be some reasons for ineffective employee training. The theory behind most of the food safety training programs is that an increase in the knowledge of food safety measures will result in improved behavior. Previous findings suggest that although food safety training might increase knowledge, it may not always translate to improved behavior (Cunha et al., 2014; Zanin et al., 2017). Only focusing on facts and knowledge is not enough to influence behavior, because it is often difficult to translate knowledge and theory into skill (Chang et al., 2003). Two important factors for evaluating the effectiveness of training are how to present the training and how is its quality (McFarland et al., 2019).

The results of a systematic review and meta‐analysis on the effectiveness of food safety education interventions for consumers in the developed countries showed that different educational interventions were effective in improving the consumers’ food safety outcomes (Young et al., 2015). The results of a study among 200 chefs and catering managers in Ireland indicated that although they were aware of basic knowledge in order to deliver safe food according to the law, they still needed extra training to increase their knowledge toward implementing food safety effectively (Bolton et al., 2008).

The findings of another systematic review in Canada revealed that routine intervention education of food service premises is effective in reducing the risk of foodborne illnesses and improving the knowledge and practices of food handlers. Furthermore, it was reported that community‐based education programs can increase public knowledge of food safety (Campbell et al., 1998). In summary, there is some evidence indicating the effectiveness of multiple public health interventions to ensure food safety. It is important to emphasize the need for ongoing high‐quality research to inform decision‐makers about health and food safety.

For evaluating the quality of training intervention in the present study, some participants commented on their feedback and preference for a longer training session as this would enable them to introduce some active learning elements to the training (e.g., small group work around communication with health and food safety for customers, a feature which may reinforce learning and improve participant satisfaction). The training event improved the participants’ knowledge, attitude, and practice, while they were, generally, satisfied. Also, the food handlers' suggestions for training needs included appropriate involvement of managers, fewer trainees per course, camera installation for observation of their practices, and longer course duration in the appropriate location in their workplace.

It seems that the restaurant industry struggled to recruit quality hires in the pandemic, so restaurant managers would be wise to employ skilled food handlers. Accordingly, restaurant managers should pay more attention to the prevention skills of COVID‐19 during the job interviews to recruit food handlers. After hiring them, it is recommended to develop regular courses to monitor, qualify, and update their information through motivating and encouraging them to participate in these courses. A study in 2021 in Iran recommended that the governments should support the restaurant managers to increase their staffs’ knowledge and attitude by online intensive update courses that can lead to desirable practice for prevention of COVID‐19 in the community. Also, the restaurant industry should prepare for future crises. The managers must follow all instructions issued by the authorities in order to reduce workplace risk to the lowest reasonably practicable level by taking preventative measures (Mohammadi‐Nasrabadi et al., 2020).

### 4.1 Future research

In closing, future research is needed as the COVID‐19 pandemic continues, the restaurant industry into the type of continuous training and how to implementation as a new normal.

Food safety training should be practical and easy to apply. The scope of this training should encompass all society sectors. Properly improving the knowledge, attitude, and practice of food handlers through the health and food safety training system can minimize COVID‐19 outbreak before leading to a public health emergency.

Furthermore, restaurateurs could work with governmental health agencies to provide, for example, an official certificate of health and food safety. These certificates could further boost consumer confidence (Brizek et al., 2020). The results of this paper can be important to share and outline our recommendations to guide future training.

## 5 LIMITATIONS AND STRENGTHS

The main limitation of the present study was that due to the long duration of the COVID‐19 pandemic, the majority of the restaurants were closed and most of the managers were not willing to cooperate with this study. So instead of selecting a control group, the main group for intervention was controlled in order to not attend any other similar training program during the study course. Another limitation was that the practical activities could not be observed and the food handlers’ practice was self‐reported.

The strength of this study was that all restaurant managers and food handlers were interested and persistent because of their own health and the survival of their restaurant, so they had a good cooperation with the research team.

## 6 CONCLUSION

In conclusion, although there is no direct and credible evidence that COVID‐19 is a foodborne disease, contact with food cannot be considered as completely safe in the context of an ongoing pandemic. The present research results regarding the effect of implementing a training intervention on health and food safety during the COVID‐19 pandemic showed that the mean knowledge level of food handlers was good before and after training, their attitude level changed from negative to positive, and their practice changed from acceptable to desirable. It seems that improving the health and food safety educational intervention of food handlers can minimize the COVID‐19 outbreak and improve the status of restaurants; this can be an effective step in the health of the community. Overall, the study findings indicated that there is scope for improvement of health and food safety training in food handlers. Researchers, food safety communicators, unions, media, and all other related sectors should work toward educating the food handlers to advance their health and food safety training. Public health authorities should prioritize food handlers in restaurants for training courses, especially during the COVID‐19 pandemic. Finally, it is recommended that policymakers use the results of this study to design an online system of continuous training for health and food safety and inspection for restaurants.

## CONFLICTS OF INTEREST

All authors declared no personal or financial conflicts of interest.

## AUTHOR CONTRIBUTION

**Fatemeh Mohammadi‐Nasrabadi:** Formal analysis (equal); Methodology (equal); Writing‐review & editing (equal). **Yeganeh Salmani:** Data curation (equal); Methodology (equal); Writing‐original draft (equal); Writing‐review & editing (equal). **Fatemeh Esfarjani:** Conceptualization (lead); Data curation (equal); Methodology (equal); Supervision (equal); Writing‐original draft (equal).

## ETHICAL APPROVAL

This study was conducted according to the guidelines laid down in the Declaration of Helsinki, and all procedures involving the research study participants were approved by the Research Council of National Nutrition and Food Technology Research Institute. Written informed consent was obtained from all stockholders. Also, they signed a written informed consent form before the interview, and explicit permission was sought from all of them for audio‐taping.

Ethical issues (including plagiarism, informed consent, misconduct, data fabrication and falsification, double publication and submission, redundancy, etc.) have been completely observed by the authors.
